# Inhibition and reversal of a TGF-β1 induced myofibroblast phenotype by adipose tissue-derived paracrine factors

**DOI:** 10.1186/s13287-024-03776-3

**Published:** 2024-06-13

**Authors:** S. Higginbotham, V. L. Workman, A-V. Giblin, N. H. Green, D. W. Lambert, V. Hearnden

**Affiliations:** 1 Department of Materials Science and Engineering, University of Sheffield, Sheffield, UK; 2 School of Clinical Dentistry, University of Sheffield, Sheffield, UK; 3 Newcastle Fibrosis Research Group, Institute of Cellular Medicine, Newcastle University, Newcastle upon Tyne, UK; 4 Department of Plastic Surgery, Sheffield Teaching Hospitals NHS Foundation Trust, Sheffield, UK; 5 INSIGNEO Institute for in silico Medicine, University of Sheffield, Sheffield, UK

**Keywords:** Scarring, Autologous fat grafting, Myofibroblasts, Transforming growth factor β-1, Adipose-derived stromal cells

## Abstract

**Background:**

Hypertrophic scarring results from myofibroblast differentiation and persistence during wound healing. Currently no effective treatment for hypertrophic scarring exists however, autologous fat grafting has been shown to improve scar elasticity, appearance, and function. The aim of this study was to understand how paracrine factors from adipose tissues and adipose-derived stromal cells (ADSC) affect fibroblast to myofibroblast differentiation.

**Methods:**

The transforming growth factor-β1 (TGF-β1) induced model of myofibroblast differentiation was used to test the effect of conditioned media from adipose tissue, ADSC or lipid on the proportion of fibroblasts and myofibroblasts.

**Results:**

Adipose tissue conditioned media inhibited the differentiation of fibroblasts to myofibroblasts but this inhibition was not observed following treatment with ADSC or lipid conditioned media. Hepatocyte growth factor (HGF) was readily detected in the conditioned medium from adipose tissue but not ADSC. Cells treated with HGF, or fortinib to block HGF, demonstrated that HGF was not responsible for the inhibition of myofibroblast differentiation. Conditioned media from adipose tissue was shown to reduce the proportion of myofibroblasts when added to fibroblasts previously treated with TGF-β1, however, conditioned media treatment was unable to significantly reduce the proportion of myofibroblasts in cell populations isolated from scar tissue.

**Conclusions:**

Cultured ADSC or adipocytes have been the focus of most studies, however, this work highlights the importance of considering whole adipose tissue to further our understanding of fat grafting. This study supports the use of autologous fat grafts for scar treatment and highlights the need for further investigation to determine the mechanism.

**Supplementary Information:**

The online version contains supplementary material available at 10.1186/s13287-024-03776-3.

## Introduction

Hypertrophic dermal scarring is a debilitating condition characterised by excessive collagen and extracellular matrix (ECM) deposition [1]. During healthy wound healing, regeneration and remodelling results in scars which closely resemble the surrounding skin. During hypertrophic scar formation this process is disrupted and the inflammatory phase is prolonged resulting in raised and rigid scars which remain within the confines of the originally injured area. Hypertrophic scars can be painful, itchy, tight, cosmetically undesirable and can limit movement but there is currently no effective cure. This can significantly affect a patient’s mental health and quality of life [2, 3]. Current treatment options for hypertrophic scarring including surgery, topical agents and physical therapy have shown limited success and result in minimal improvements in scar outcomes [1, 4, 5].

In recent years autologous fat grafting has generated significant clinical interest for the treatment of hypertrophic scarring. Subcutaneous injections of adipose tissue have been shown to regenerate dermal tissue and improve skin function and appearance [6, 7]. Adipose tissue is composed of adipocytes and a heterogeneous cell population called the stromal vascular fraction (SVF) [8], within which there is a stem cell-like population known as adipose-derived stromal cells (ADSC) [9]. ADSCs are thought to be partly responsible for the improvement in scarring seen from autologous fat grafting [7, 10, 11], however, there is growing interest into how the other components of adipose tissue contribute to its regenerative effects [12, 13].

A large number of ADSCs can be isolated and expanded from adipose tissue and factors secreted from ADSCs have been shown to reduce inflammation [14], increase angiogenesis [15], and inhibit the differentiation of fibroblasts into myofibroblasts [16]. Therapies delivering ADSC alone or in combination with lipoaspirate are currently in clinical trials but the regulatory, financial and logistical challenges associated with autologous cell therapy will limit its accessibility and adoption [17]. Autologous fat grafting is a more attractive option for hypertrophic scar therapy as adipose tissue is readily accessible through liposuction, can be administered in a single surgical procedure and it is well accepted by patients and surgeons.

Myofibroblasts differentiate from fibroblasts following the release of Transforming Growth Factor (TGF-β1) during wound healing [18] and are a key cell type involved in scar formation and maturation [19]. Myofibroblast populations are normally cleared through apoptosis following scar resolution [20], however in hypertrophic scarring myofibroblast populations persist [21]. Myofibroblasts are characterized by increased expression of alpha smooth muscle actin (α-SMA), increased production of extracellular matrix (ECM) components and a reduction in matrix metalloprotease production. As a result the ECM found in hypertrophic scars is stiffer and less organised compared to surrounding healthy tissues [1, 22, 23]. Fibroblast to myofibroblast differentiation can be induced with TGF-β1 treatment and this is well established as a simple but valuable model to study dermal scarring in vitro [24].

It has been proposed that inhibiting myofibroblast differentiation or reducing the myofibroblast population in scars could alter scar phenotype and lead to improved patient outcomes [25]. Previous studies have investigated the role of secreted factors from ADSCs [16] and adipocytes on myofibroblast behaviour [13] and have both shown that paracrine factors were able to inhibit myofibroblast differentiation and reduce collagen production. However, these studies with monocultures fail to fully capture the clinical situation where a heterogeneous population of cells and their associated ECM are injected during autologous fat grafting. A greater understanding of how all components of adipose tissue act in combination is needed to support and further develop the clinical use of autologous fat grafting. To the best of our knowledge there are no previous studies which have investigated the role of secreted factors from whole adipose tissue on myofibroblast behaviour.

This study aimed to determine whether paracrine factors from adipose tissue, ADSC or lipid extracted from adipose tissue could inhibit and/or reverse myofibroblast differentiation in an in vitro model of dermal scarring. Furthermore, this study aimed to improve our understanding of the mechanisms responsible for the positive clinical effects seen following fat grafting for scar revision.

## Materials and methods

### Human tissue isolation and lipid extraction

Human adipose tissue and skin, waste from routine surgery, was collected with informed consent following a protocol approved by the NHS research ethics committee (references 15/YH/1077 and 21/NE/0115). Tissue was received either as solid adipose tissue with skin or as lipoaspirate. Solid adipose tissue was minced with a scalpel, removing large blood vessels and connective tissue, until a consistency matching lipoaspirate was achieved. Both types of tissue were washed in phosphate buffered saline (PBS (Thermo Fisher Scientific, UK)) and centrifuged at 1200*g* for 3 min to remove drugs from surgery, excess lipid and blood.

Lipid, for use as a control to rule out the influence of free lipid from adipose tissue, was extracted from fat by emulsifying the tissue as in Tonnard et al. [11]. This emulsion was then centrifuged at 2000*g* for 3 min and lipid was removed with a Pasteur pipette and stored at 4 °C until used on cells.

### Cell isolation and culture

Human dermal fibroblasts (HDF) and human scar fibroblasts (HSF) were isolated from normal human skin (HDF) and from scarred skin as defined by surgeons (HSF), using collagenase digestion, as previously described [26]. The dermis and epidermis were first separated from the underlying hypodermis using a skin graft knife and keratinocytes were removed by incubating in a 0.1% (w/v) solution of Difco™ Trypsin 250 (Merck, UK) overnight at 4 °C. Keratinocytes were scraped from the surface and the remaining dermis was finely minced using a scalpel. The minced dermis was incubated in 0.05% (w/v) Collagenase A extracted from Chlostridium histolyticum (Merck, UK) at 37 °C for 18 h. The digested dermis was then centrifuged and placed into tissue culture flasks to allow HDF adhesion. Human scar fibroblasts (HSF) were isolated as described above from skin which had been classified as scar tissue from the surgeon excising the tissue. Fibroblasts were maintained in a solution of D-MEM (Dulbecco’s-modified Eagle’s medium (Merck, UK)) containing 10% foetal calf serum (Pan- Biotech, UK), 200 mM glutamine, 10,000 units/ml penicillin, 10 mg/ml streptomycin, and 250 μg/ml amphotericin B solution (all Merck, UK) and passaged with Trypsin–EDTA (Merck, UK) upon reaching 90% confluency. Fibroblasts were not cultured beyond passage 7.

ADSC were isolated from washed solid adipose tissue or lipoaspirate. The adipose tissue was incubated in a collagenase I solution (Hank’s balanced salt solution (Thermo Fisher Scientific, UK) containing 0.1% w/v collagenase l, 0.1% w/v bovine serum albumin, 10,000 units/ml penicillin and 10 mg/ml streptomycin (all Merck, UK)) at 37 °C for 40 min and inverted every 10 min. This mixture was centrifuged at 257*g* for 8 min and the supernatant layers removed. The resulting pellet was resuspended in 2% MesenPRO™ RS media (MesenPRO RS™ basal medium containing 2% MesenPRO RS™ growth supplement (both Thermo Fisher Scientific, UK)) and transferred to a tissue culture flask. After 24 h, the media was replaced, and the flask washed with PBS. ADSCs were maintained in 2% MesenPRO™ RS media and were used up to passage 6. All cells were tested for mycoplasma using PlasmoTest™ (InvivoGen, UK) once a month.

### Generation of conditioned media

Adipose tissue conditioned medium was generated by incubating 0.1 g/ml of minced fat or lipoaspirate in serum free MesenPRO™ RS media for 72 h in a 37 °C, 5% humidified CO_2_ incubator. ADSC conditioned medium was generated by seeding 4500 ADSC/cm^2^ for 24 h in serum free MesenPRO™ RS media (15,000 ADSC/ml). Following this, the media was replaced and incubated for 72 h in a 37 °C, 5% humidified CO_2_ incubator. After 72 h, conditioned media was centrifuged at 2645*g* for 8 min and where necessary adipose tissue was removed. Conditioned media from ADSC or adipose tissue was then filtered through 100 μm filters (PluriSelect, UK) and stored at −80 °C until use. For lipid conditioned medium, serum free MesenPRO™ RS media was applied to cells at the beginning of an experiment and 0.05 ml of free lipid per ml of media was added.

### Myofibroblast differentiation

To induce myofibroblast differentiation, HDF or HSF (seeded at a density of 3000 cells/cm^2^) were treated with 5 ng/ml TGF-β1 (Peprotech, UK) in serum free MesenPRO RS™ media. Prior to TGF-β1 treatment, HDF or HSF were cultured in serum free MesenPRO RS™ media for 24 h.

### Gene expression analysis

RNA was extracted from HDF or HSF using a RNeasy Plus Mini kit (Qiagen, UK) and cDNA generated using a High-Capacity RT PCR kit (Thermo Fisher Scientific, UK). SYBR green qPCR (PCR Biosystems, UK) was carried out with the primers detailed in Table 1 (Qiagen, UK). Fold changes in gene expression were calculated using the 2^–∆∆Ct^ method [27] and normalised to RNU6-1 expression.

**Table 1:** Primers used for qPCR

Name	Primer	Forward	Reverse
U6 small nuclear 1	RNU6-1	5′-CTCGCTTCGGCAGCACA-3′	5′-AACGTTCACGAATTTGCGT-3′
Smooth muscle actin	⍺-SMA	5′-GAAGAAGAGGACAGCACTG-3′	5′-TCCCATTCCCACCATCAA-3′
Collagen I	COL1-A1	5′-GTGGCCATCCAGCTGACC-3′	5′-AGTGGTAGGTGATGTTCTGGGAG-3′
Fibronectin	FN1-EDA	5′-TGGAACCCAGTCCACAGCTATT-3′	5′-GTCTTCTCCTTGGGGGTCACC-3′

### Protein immunoblotting

To quantify protein expression HDF were lysed with protein lysis buffer (radioimmunoprecipitation buffer (Universal Biologics, UK) containing one complete mini protease inhibitor cocktail tablet (Merck) and 1 μl/ml Benzonase® nuclease (Merck, UK)). Total protein quantity was calculated using a Pierce BCA assay (Thermo Fisher Scientific, UK). SDS-PAGE and western blotting were then carried out using anti-GAPDH and anti-α-SMA antibodies (Table 2). Antibodies were diluted in tris-buffered saline (6.05 g/L tris(hydroxymethyl)aminomethane, 8.76 g/L sodium chloride (both VWR), 1 ml/L Tween 20 (Merck, UK), in deionised water at pH 7.6) containing 5% w/v dry milk powder (Generon, UK). Protein blots were imaged on a C-Digit scanner (Li-Cor Biosciences, UK) and protein expression was quantified using Fiji software [28].

**Table 2:** Antibodies used in western blotting

Antibody	Species	Size (kDa)	Dilution	Supplier	Product code
Anti-α-SMA	Rabbit	42	1:10,000	Abcam	Ab124964
Anti-GAPDH	Mouse	37	1:5000	Proteintech	60,004–1-lg

*α-SMA* α-smooth muscle actin, *GAPDH* glyceraldehyde 3-phospate dehydrogenase

### Immunofluorescence staining

For immunofluorescent images, HDF/HSF were seeded onto sterilised coverslips at a density of 3000 cells/cm^2^ and treated with TGF-β1 and/or conditioned media. Following treatment, cells were fixed with 100% methanol and permeabilised with 1% Triton-X (Thermo Fisher Scientific, UK). Cells were either mono-stained for α-SMA or dual-stained for α-SMA and Ki-67 and nuclei were stained with 4′,6-diamindino-2-phenylindole (DAPI). All antibodies were added in 1% BSA (Merck, UK) in PBS. For mono-stained cells an α-SMA-fluorescein isothiocyanate (FITC) conjugated antibody (Table 3) was used and slides examined on a IX73 inverted Olympus fluorescent microscope with images taken on a Retiga 6000 camera (Teledyne Photometrics, UK). Dual stained cells were stained using primary α-SMA and Ki-67 antibodies alongside Alexa Fluor® secondary antibodies. These cells were then examined on an upright Zeiss LSM510 Meta confocal microscope.

**Table 3:** Antibodies used in fluorescent imaging

Antibodies and stains	Species	Dilution	Supplier	Product code	Excitation
Ki-67	Rabbit	1:1000	Abcam	Ab15580	–
α-SMA	Mouse	1:1000	Abcam	Ab7817	–
Anti-rabbit Alexa Fluor® 647 conjugated	Donkey	1:1000	Abcam	Ab150063	HeNe2 (633 nm)
Anti-mouse Alexa Fluor® 555 conjugated	Goat	1:1000	Abcam	Ab150118	HeNe1 (543 nm)
α-SMA-FITC conjugated	Mouse	1:500	Merck	F3777	LED, 488 nm filter used
DAPI	–	1:1000	Merck	D8417	Argon 2 (458 nm)

Abbreviations used are α – smooth muscle actin (α-SMA), Fluorescein Isothiocyanate (FITC), 4′,6-Diamindino-2-Phenylindole (DAPI)

Cell nuclei were counted using Fiji software and the number of cells positive for ⍺-SMA was recorded. A cell was considered α-SMA positive if the nuclei was overlaid on stained α-SMA fibres. For each sample, three images were taken, and the cell counts averaged. There was between 50 and 200 cells per field of view on average. The only exception to this were the images in Fig. 4E–N. These images were taken at a lower magnification and contained between 200 and 1000 cells, thus only one image was taken and quantified per sample. Brightness was increased on images to aide counting, this change was applied uniformly on all images in each respective repetition on Fiji.

### Hepatocyte growth factor quantification

Total protein concentration in adipose tissue conditioned media and ADSC conditioned media was calculated via a Pierce BCA assay. Conditioned media samples were then diluted with serum free MesenPRO RS™ media such that all samples contained an equal concentration of total protein. For each measurement conditioned media from four tissue/cell samples from different patients (unmatched) were pooled together and a hepatocyte growth factor (HGF) enzyme-linked immunosorbent assay (ELISA (Merck, UK)) was carried out as per the manufacturer’s instructions.

### HGF and foretinib treatment

For experiments involving HGF and/or foretinib treatment HDF were seeded at a density of 3000 cells/cm^2^ and were cultured in serum free MesenPRO RS™ media for 24 h prior to treatment. HDF were then treated with 5 ng/ml of TGF-β1 alongside 40 ng/ml of recombinant human HGF (Peprotech, UK; reconstituted as per the manufacturer’s instructions) and/or 1 nM foretinib (Cambridge Biosciences, UK; resuspended in DMSO) in serum free MesenPRO RS™ media.

### Statistical analysis

Data are represented as mean ± standard deviation (SD). Data was analysed using GraphPad Prism version 9.5.0 for Windows (GraphPad Software, Boston, Massachusetts USA, www.graphpad.com). Details of statistical tests used are included in figure legends and values of *p* < 0.05 were considered statistically significant.

## Results

### Application of TGF-β1 induced a scar-like phenotype in HDF

**Fig. 1:**
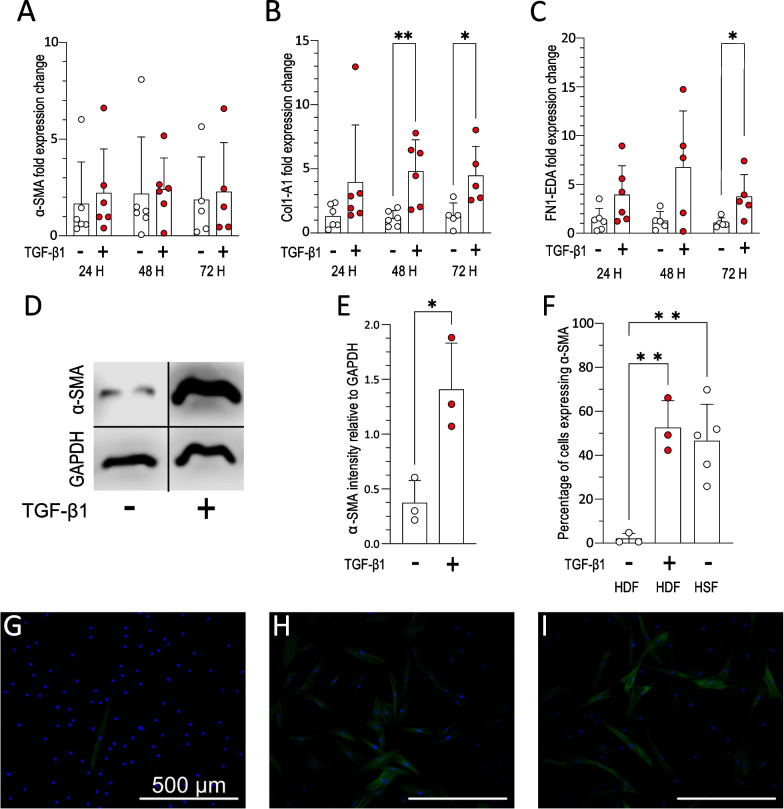
Characterisation of TGF-β1 dependant myofibroblast differentiation. **A**–**C** Reverse transcription quantitative PCR (RT-qPCR) of **A** α-SMA, **B** collagen 1 and **C** fibronectin, mRNA expression at 24, 48, and 72 h with/without TGF-β1 treatment (*n* = *3, N* = *5/6*)*.* **D** Representative immunoblotting of α-SMA protein in HDF treated with/without TGF-β1. Images are cropped for visual ease; full length blots are presented in Additional file 1: Figure S1. **E** Quantification of α-SMA protein from immunoblotting images (*n* = *1, N* = *3*). **F** Myofibroblast differentiation was assessed by the percentage of the fibroblasts positive for α-SMA from immunofluorescence images, three images were taken for each repeat and averaged (*n* = *3, N* = *3/5*). **G**–**I** Representative immunofluorescence images of **G** untreated HDF, **H** TGF-β1 treated HDF, **I** untreated HSF stained for α-SMA fibres (green) and counter stained for nuclei (blue). Scale bar = 500 µm. To assess significance, Mann–Whitney tests were carried out on data from figures B and C as data was not normally distributed. Where data was normally distributed an un-paired t-test was used (figure A and E), and an ordinary one-way ANOVA in figure F. Error bars show standard deviation, *p < 0.05; ** *p* < 0.01

Markers of myofibroblast differentiation were examined following TGF-β1 treatment of HDF. There was no significant change in α-SMA at any time point tested (Fig. 1A). Collagen mRNA expression significantly increased after 48 and 72 h of TGF-β1 treatment and fibronectin mRNA expression was significantly increased after 72 h of TGF-β1 treatment (Fig. 1B, C). Immunoblotting was carried out on lysate from HDF and TGF-β1 treated HDF which showed α-SMA protein expression was higher following 72 h of TGF-β1 treatment (Fig. 1D–E). Immunocytochemistry revealed that there was a higher proportion of α-SMA positive cells following 72 h of TGF-β1 treatment compared to untreated HDF cells (Fig. 1F–H).

To validate this in vitro model of scarring, TGF-β1 treated fibroblasts were compared to human scar fibroblasts (HSF) isolated from established scar tissue (Fig. 1F, I). The proportion of α-SMA positive cells was comparable between HSF (47% ± 16.7) and TGF-β1 treated HDF (53% ± 12.3) and was significantly higher than untreated HDF (2% ± 2.4). Together these data showed that 72 h of TGF-β1 treatment was able to induce myofibroblast differentiation in HDF and that α-SMA immunocytochemistry could be used as a reliable method to quantify differentiation in a cell model which resembled cells isolated from established scars.

### Adipose tissue conditioned medium inhibits TGF-β1 dependant myofibroblast differentiation in HDF

**Fig. 2:**
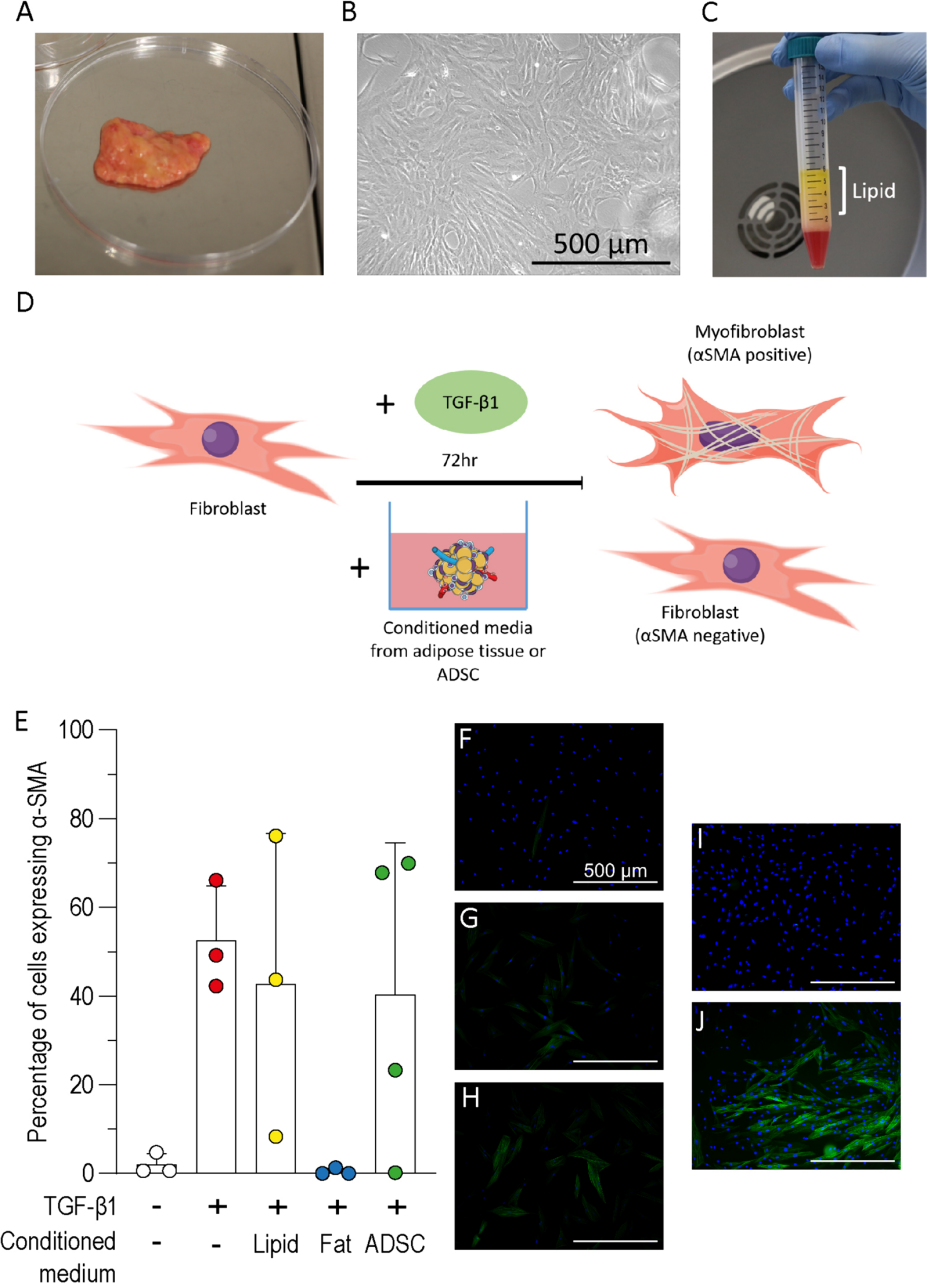
Inhibition of TGF-β1 dependant myofibroblast differentiation by adipose tissue conditioned medium. Conditioned media was prepared using **A** minced adipose tissue, **B** ADSCs or **C** lipid extracted from lysed adipose tissue (marked by white bar). **D** Schematic of experimental conditions used to test the ability of conditioned media to inhibit TGF-β1 induced myofibroblast differentiation. Cell culture medium was conditioned with adipose tissue for 72 h and then used to treat HDF alongside TGF-β1 for 72 h. The extent of myofibroblast differentiation was then quantified using α-SMA immunofluorescence as a marker of myofibroblast differentiation. **E** Myofibroblast differentiation was quantified by the percentage of HDF positive for α-SMA from immunofluorescence images. Three images were taken for each repeat and cell counts were averaged (n = 3, N = 3 or 4). **F**–**J** Representative immunofluorescence images of HDF stained for α-SMA fibres (green) and counter stained for nuclei (blue) following treatment with TGF-β1 and conditioned media. Untreated HDF (**F**), HDF treated with TGF-β1 (**G**), lipid conditioned medium and TGF-β1 (**H**), adipose tissue (fat) conditioned medium and TGF-β1 (**I**), and ADSC conditioned medium and TGF-β1 (**J**). Scale bar = 500 µm. Error bars show standard deviation. A Kruskal–Wallis test with Dunn’s multiple comparison post-hoc test was used in figure E and no significant differences were found

Adipose tissue (fat) and ADSC isolated from patient tissue was used to condition cell culture medium (Fig. 2A, B). Unconditioned media and media conditioned with lipid extracted from lysed adipose tissue (with no viable cells) were used as controls (Fig. 2C). HDF were treated with fat, ADSC or lipid conditioned medium alongside TGF-β1 for 72 h and α-SMA protein expression were measured (Fig. 2D).

When treated with TGF-β1, approximately half of the HDF stained positively for α-SMA. The addition of lipid or ADSC conditioned medium had no significant effect on the proportion of α-SMA positive cells however, treatment with adipose tissue conditioned medium led to a dramatic reduction in α-SMA positive cells (Fig. 2E). Representative images of α-SMA labelled cells for each condition are shown in (Fig. 2F–J). Together this data implies that paracrine factors from fat can inhibit TGF-β1 dependent myofibroblast differentiation in vitro.

### The inhibition of myofibroblast differentiation is not driven by HGF

**Fig. 3:**
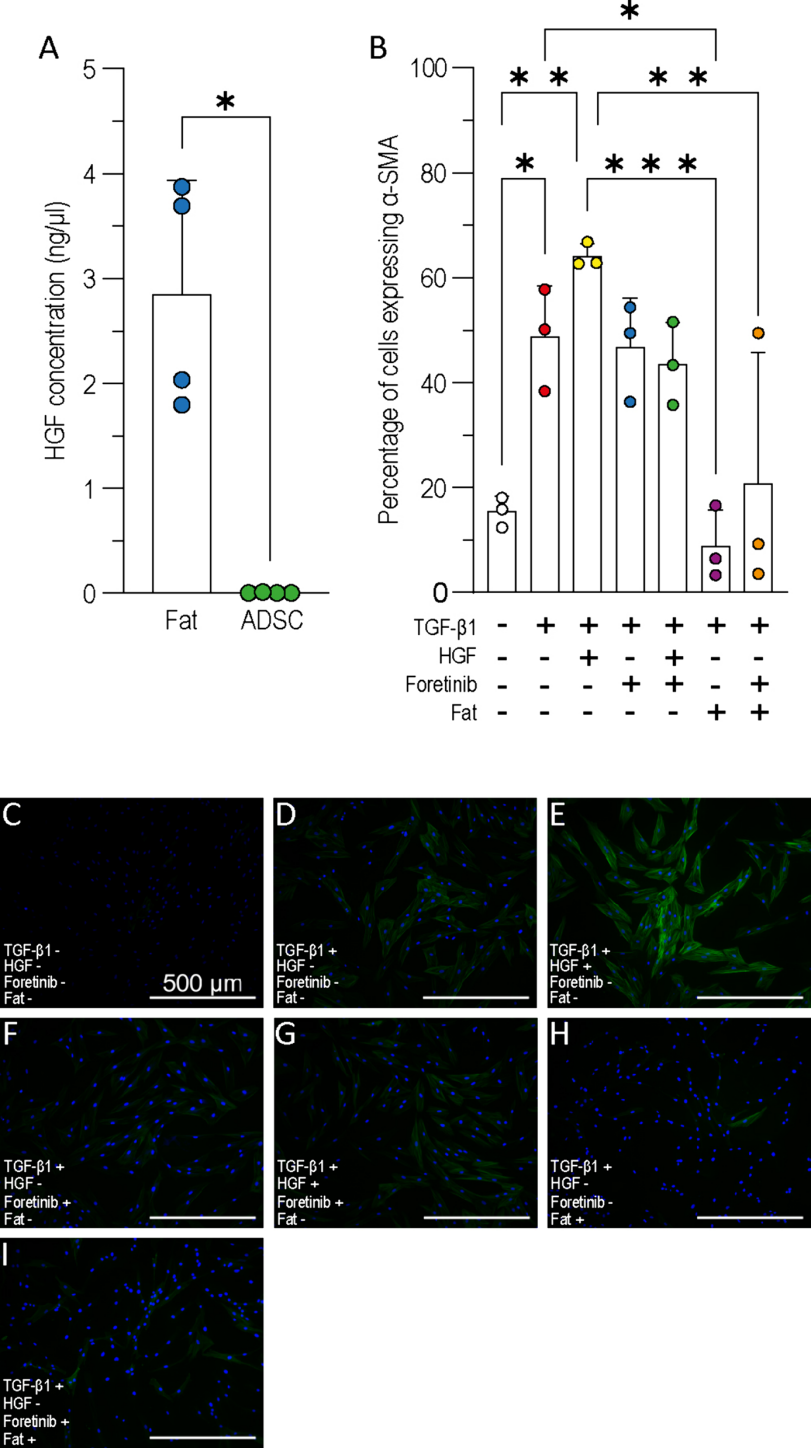
The effect of HGF and foretinib on TGF-β1 dependant myofibroblast differentiation. **A** HGF concentration in conditioned media quantified via ELISA (*n* = *2, N* = 4)*.* **B** Myofibroblast differentiation quantified by the percentage of α-SMA positive HDF following 40 h TGF-β1 treatment alongside HGF/foretinib/adipose tissue conditioned medium (*n* = 3*, N* = 3)*.* **C**–**I** Representative immunofluorescence images of **C** untreated HDF, **D** HDF treated with TGF-β1, **E** TGF-β1 and HGF, **F** TGF-β1 and foretinib, **G** TGF-β1, HGF, and foretinib, **H** TGF-β1 and adipose tissue (fat) conditioned medium, **I** TGF-β1, foretinib, and adipose tissue conditioned medium, stained for nuclei (blue) and α-SMA fibres (green). Scale bar = 500 μm. A Mann–Whitney test was used to assess significance in figure A and an ordinary one-way ANOVA with Tukey’s multiple comparisons post-hoc test was used for figure B. Error bars show standard deviation, *p < 0.05; **p < 0.01; ***p < 0.001

Analysis of adipose tissue conditioned media identified a number of proteins which were expressed in higher concentrations compared to ADSC conditioned media [29]. Of particular interest was HGF which has previously been reported to inhibit the TGF-β1 pathway in fibrosis and which acts primarily through the cMet receptor [30–32]. The concentration of HGF in adipose tissue conditioned medium was significantly higher than in ADSC conditioned medium (Fig. 3A).

To test whether the myofibroblast inhibition seen with adipose tissue conditioned media treatment was a result of HGF, HDF were treated with TGF-β1, HGF and foretinib (a cMet inhibitor). Treatment was applied for 40 h (the half-life of foretinib [33] and the proportion of α-SMA positive cells was quantified from fluorescent immunocytochemistry images (Fig. 3B–I). As shown previously, treating HDF with TGF-β1 led to a significantly higher proportion of α-SMA positive cells compared to untreated HDF (Fig. 3B–D). When HGF was applied alongside TGF-β1 64.1% (± 2.4) of cells were positive for α-SMA compared to 48.7% (± 9.8) treated with TGF-β1 but without HGF (Fig. 3B, D, E). While this was not a significant difference this increase was not seen when cells were treated with HGF and blocked with fortinib (46.1% ± 15.6, Fig. 3B, G). The addition of fortinib with TGF-β1 did not affect the proportion of α-SMA cell (Fig. 3B, F). Applying adipose tissue conditioned medium alongside TGF-β1 lowered the proportion of α-SMA positive HDF (8.8% ± 6.9, Fig. 3H) but the addition of foretinib alongside adipose tissue conditioned medium did not significantly alter the proportion of α-SMA positive cells (20.8% ± 25.0, Fig. 3B, I). Together, this data demonstrates that HGF alone is not responsible for the inhibition of TGF-β1 dependent myofibroblast induction seen in our previous experiments.

### Adipose tissue conditioned medium can reverse TGF-β1 induced myofibroblast differentiation

**Fig. 4:**
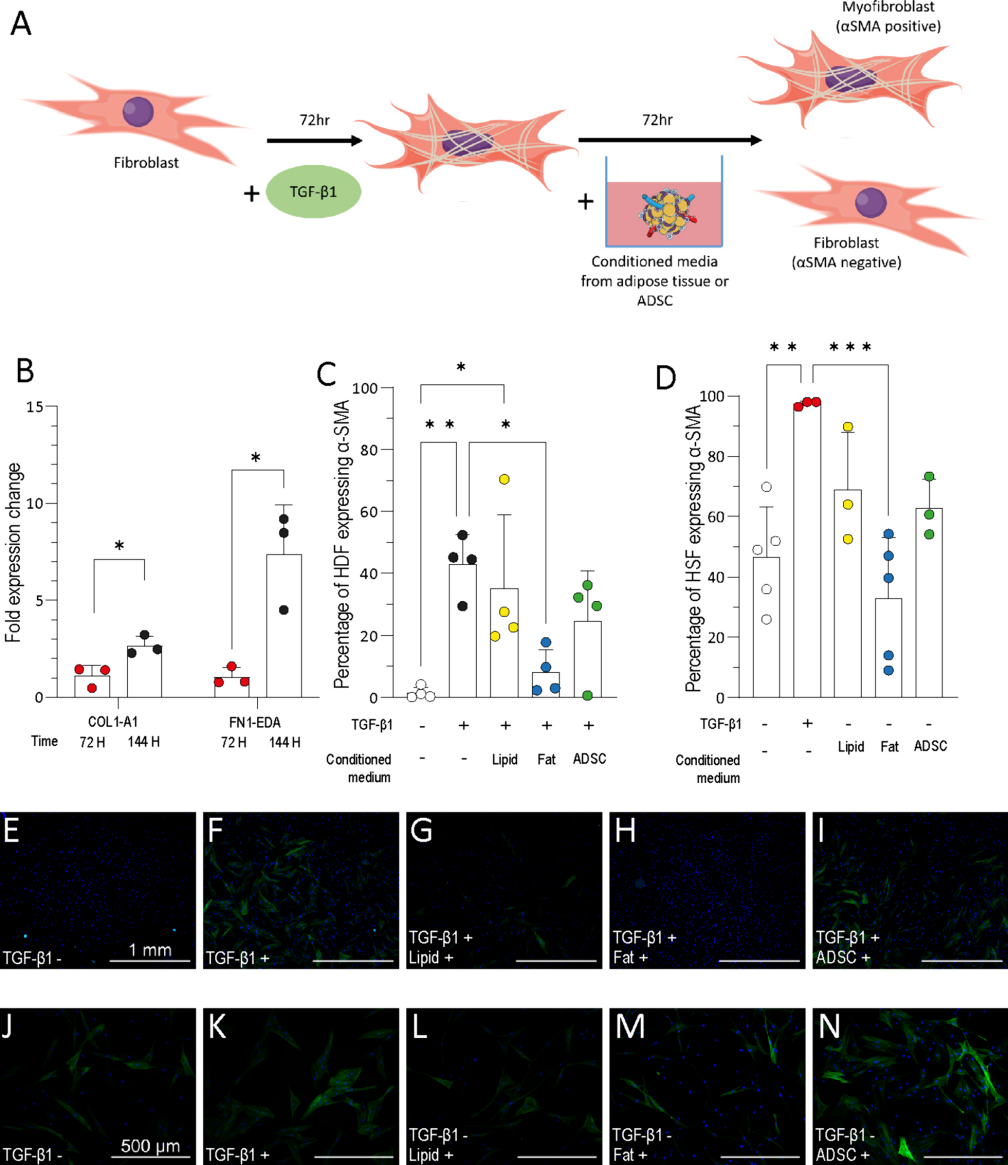
Reversal of TGF-β1 dependant myofibroblast differentiation by adipose tissue conditioned medium. **A** Schematic of experimental conditions used to test the ability of conditioned media to reverse myofibroblasts differentiation. HDF were differentiated to myofibroblasts with TGF-β1 incubation for 72 h. Following this, media was replaced and cells were treated with conditioned medium for 72 h and markers of myofibroblast phenotype were investigated. **B** RT-qPCR of collagen 1 and fibronectin mRNA in HDF after 72 h of TGF-β1 treatment (72 H) followed by 72 h of serum free media treatment (144H) (*n* = 3*, N* = 3). **C** Myofibroblast differentiation was assessed by the percentage of HDF positive for α-SMA following 72 h TGF-β1 treatment followed by 72 h of conditioned media treatment (*n* = 1*, N* = 4). **D** Myofibroblast differentiation was quantified in HSF treated with conditioned medium for 72 h as represented by the percentage of HSF positive for α-SMA from immunofluorescence images, three images were taken for each repeat and counts averaged (*n* = 3,* N* = 3 or 5). **E**–**I** Representative immunofluorescence images of TGF-β1 differentiated HDF stained for nuclei (blue) and α-SMA fibres (green). Untreated (**E**), TGF-β1 treated (**F**), lipid conditioned medium and TGF-β1 (**G**), adipose tissue (fat) conditioned medium and TGF-β1 (**H**), and ADSC conditioned medium and TGF-β1 (**I**). Scale bar = 1 mm. **J**–**N** HSF treated with conditioned media stained for nuclei (blue) and α-SMA fibres (green). Untreated (**J**), TGF-β1 treated (**K**), lipid conditioned medium (**L**), adipose tissue (fat) conditioned medium (**M**), and ADSC conditioned medium (**N**). Scale bar = 500 µm. Unpaired t-tests were used to assess significance in figure **B** and ordinary one-way ANOVA was used for figures **C**, **D**. Error bars show standard deviation, *p < 0.05; **p < 0.01, ***p < 0.0001

To test the ability of conditioned media to reverse fibroblast to myofibroblast differentiation, HDF were treated with TGF-β1 for 72 h. Following this treatment, TGF-β1 treated cells were cultured with adipose tissue conditioned medium or ADSC conditioned medium for a further 72 h (Fig. 4A).

To ensure the myofibroblast differentiation state was not reversed following the withdrawal of TGF-β1, collagen and fibronectin mRNA expression was quantified 72 h after the withdrawal of TGF-β1 treatment. Collagen and fibronectin mRNA expression was significantly increased 72 h after TGF-β1 was removed (Fig. 4B). Cells treated with TGF-β1 for 72 h followed by control medium for 72 h also maintained a high proportion of α-SMA positive cells (43% ± 9.6) (Fig. 4C), which was similar to the proportion of α-SMA positive cells after 72 h of TGF-β1 treatment (53% ± 12.3, Fig. 1F). This demonstrated spontaneous myofibroblast de-differentiation was not occurring.

HDF cultured for 144 h with control media maintained a low proportion of α-SMA positive cells (4% ± 1.9, Fig. 4C). Cells treated with TGF-β1 and then adipose tissue conditioned medium had a significantly lower proportion of α-SMA positive cells (8% ± 7.2) compared to HDF treated with TGF-β1 and control media (43%, ± 9.6, *p* = 0.022, Fig. 4C). No effect on the proportion of α-SMA positive cells was seen in cells treated with media conditioned with ADSC or lipid compared to cells treated with TGF-β1 only (Fig. 4C).

The ability of adipose tissue conditioned medium to de-differentiate myofibroblasts was also tested on HSF. Approximately half of all HSF, isolated from established scars, were α-SMA positive (47% ± 16.7) (Fig. 4D). When HSF were treated with TGF-β1 for 72 h, the proportion of cells positive for α-SMA increased to 97% (± 0.9, *p* = 0.0054). The mean proportion of HSF expressing α-SMA following treatment with adipose tissue conditioned medium was lower than untreated HSF (33% ± 20.2) however, this decrease was not significant (*p* = 0.67). Treatment with media conditioned with ADSC or lipid did not alter the proportion of α-SMA positive HSF (63% ± 9.8). Taken together, this data suggests that factors secreted from adipose tissue can reverse TGF-β1 induced α-SMA expression in HDF but not in scar derived fibroblasts.

### Myofibroblast dedifferentiation is not a result of increased proliferation

**Fig. 5:**
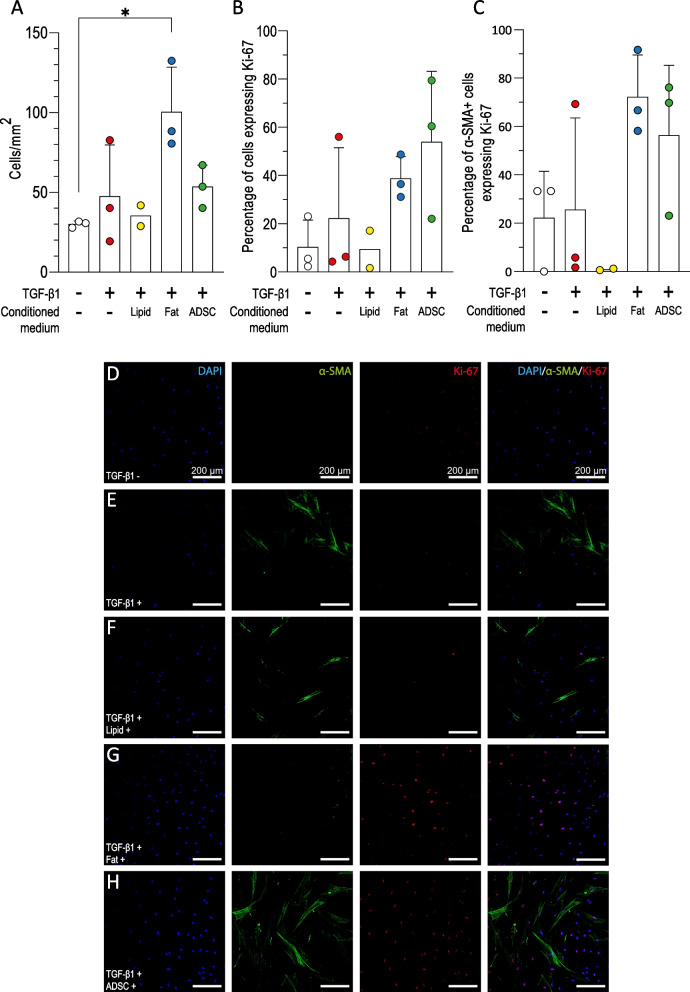
Adipose tissue and ADSC conditioned medium increases the proliferation of HDF. **A** The number of cells per mm^2^ in fluorescent images following 72 h TGF-β1 treatment followed by 72 h treatment with/without conditioned medium was quantified (*n* = 3*, N* = 2 or 3). **B** Proportion of Ki-67 positive, proliferating cells following treatment (*n* = 3*, N* = 2 or 3). **C** Quantification of proliferating myofibroblasts as represented by the percentage of cells positive for both Ki-67 and α-SMA. Three images were taken of each repeat and counts averaged (*n* = 3*, N* = 2 or 3). **D**–**H** Representative immunofluorescence images of HDF treated with TGF-β1 and then conditioned medium treatment (fat, ADSC or lipid conditioned). HDF were stained for nuclei (blue), α-SMA (green), and Ki-67 (purple). Untreated (**D**), TGF-β1 treated (**E**), lipid conditioned medium and TGF-β1 (**F**), adipose tissue (fat) conditioned medium and TGF-β1 (**G**), and ADSC conditioned medium and TGF-β1 (**H**). Scale bar = 200 µm. Ordinary, one-way ANOVA with Tukey’s multiple comparisons post-hoc test was used to test for significance. Error bars show standard deviation, *p < 0.05

Following an observation that there were a greater number of cells in samples treated with adipose tissue conditioned media we next tested whether adipose tissue conditioned medium was stimulating proliferation of TGF-β1 treated HDF. The number of cells per mm^2^ was similar for all conditions except cells treated with adipose tissue conditioned medium where there was a significant increase in cell number (Fig. 5A, *p* < 0.01). The expression of Ki-67 protein, a marker of proliferation, was quantified following treatment with TGF-β1 and conditioned media. The proportion of Ki-67 positive cells following treatment with adipose tissue conditioned medium or ADSC conditioned medium treated HDF was 39% (± 9.0) and 54% (± 29.2) respectively which was higher than the other conditions, however, this difference was not significantly different (Fig. 5B).

To determine which cell population was responsible for the overall increase in cell number the proportion of cells positive for both α-SMA and Ki-67 was quantified from dual stained samples (Fig. 5C–H). A high proportion of the α-SMA positive cells treated with TGF-β1 and fat or ADSC conditioned media were also Ki-67 positive and the overall trend was similar to that seen in the whole cell population. This shows that conditioned media from fat and ADSC was capable of stimulating proliferation in both α-SMA positive and α-SMA negative fibroblasts.

## Discussion

Autologous fat grafting has been shown clinically to improve the appearance and patient perception of hypertrophic scars [6, 7]. This novel treatment has great potential to improve the quality of life of patients living with scarring however further information is needed to understand the mechanism by which adipose tissue exerts these effects to utilize fat grafting to its full potential. The aim of this study was to investigate how factors secreted from whole adipose tissue, cultured ADSCs and lipid affected the fibroblast-myofibroblast axis, using a TGF-β1 induced differentiation model.

The TGF-β1 myofibroblast model has previously been used to study scar development in vitro [13, 16, 23, 30] and here we demonstrated that following TGF-β1 treatment approximately 50% of human dermal fibroblasts differentiated into α-SMA positive myofibroblasts, as measured through immunocytochemistry. Gene expression analysis was also conducted which revealed an increase in collagen I and fibronectin expression following TGF-β1 treatment for 72 h. However, there was no change in α-SMA gene expression at any timepoint. The expression of mRNA within a cell is transient, can change rapidly and is influenced by the surrounding environment. In contrast, the expression of α-SMA protein measured by western blot and immunocytochemistry showed a clear response to TGF-β1 treatment and demonstrated the presence of a scar relevant protein. The proportion of α-SMA positive cells, measured by immunocytochemistry was therefore used as the primary measure of myofibroblast differentiation throughout the study.

Whole human adipose tissue is highly heterogeneous with a complex mixture of cells and tissues which work synergistically to exert effects. In this study we collected factors secreted from whole adipose tissue to further our understanding of how autologous fat grafting improves scar tissue phenotype. The majority of previous studies have concentrated solely on monocultures of adipocytes or ADSC [13, 16, 34] however these are further from the clinical situation and lack the biological complexity of native tissue. Adipose tissue comprises of adipocytes (dominant by volume) along with the stromal vascular fraction (containing ADSC, endothelial and progenitor cells) as well as an immune component (e.g., tissue resident macrophages and neutrophils) [8]. In this study we were able to collect secreted factors from whole tissue which was collected following routine surgical procedures and compare it to cultured ADSC and acellular lipid from adipose tissue. Conditioned media is useful as a research tool to understand how paracrine factors from grafted adipose tissue or ADSC affect the surrounding tissues and also has potential as a cell free alternative to fat grafting for scar regeneration. The differences observed between conditioned media from adipose tissue and conditioned media from ADSC in this study demonstrates the importance of considering all components of adipose tissue.

The data presented here shows that factors secreted from adipose tissue, but not those from ADSC or lipid, were able to inhibit myofibroblast differentiation; reducing the proportion of α-SMA positive cells from 53 to 1%. These results contradict a study by Spiekman et al., which showed that ADSC conditioned medium was able to prevent the induction of a myofibroblast phenotype following treatment with TGF-β1 [16]. Spiekman et al. [16] observed lower collagen mRNA expression and contractility in fibroblasts treated with paracrine factors from ADSC but despite further investigation the mechanism remains unknown.

Differences observed between our study and previous work may be as a result of differences in experimental conditions (e.g., the use of MesenPro vs D-MEM) or biological variation between cells and tissue from different patients. Throughout this study we observed high levels of variation, which in some cases limited our ability to draw clear conclusions. This may be due to differences between the composition of factors in conditioned media from different patients or the responsiveness of HDF and HSF isolated from different individuals. In clinical practice it has been observed that some patients respond better to autologous fat grafting compared to others however relatively little is known as to why.

TGF-β1 induces myofibroblast differentiation through the SMAD pathway [35] and HGF has previously been shown to inhibit myofibroblast differentiation in a number of different fibroblast types [30, 32, 36], including with HGF derived from ADSC [32]. HGF was found in our adipose tissue conditioned medium at a higher concentration than in ADSC conditioned medium and was therefore selected for further investigation. When HGF was applied to fibroblasts in isolation a small non-significant increase in the proportion of α-SMA positive cells was observed which was not seen when HGF was blocked with fortinib. When fibroblasts were treated with adipose tissue conditioned media and fortinib there was no change compared to cells just treated with adipose tissue conditioned media. Together this demonstrated that HGF alone was not responsible for the inhibition of myofibroblast differentiation observed with adipose tissue conditioned media.

Autologous fat grafting is commonly used to treat established scars where there are existing populations of α-SMA positive myofibroblasts. Therefore, demonstrating that paracrine factors from adipose tissue can inhibition fibroblast to myofibroblast differentiation is important but to model the clinical situation more closely we next investigated whether conditioned media could reduce the proportion of α-SMA positive cells in HDF previously treated with TGF-β1 or in HSF.

Once seen as terminally differentiated [35] it is now increasingly clear that the myofibroblast phenotype is not an irreversible state and it has been shown that myofibroblasts around wound sites can differentiate into adipocytes [37–39]. We first confirmed that the α-SMA positive myofibroblast phenotype persisted in serum free medium for 72 h despite the cessation of TGF-β1 treatment (in contrast to a previous study which showed TGF-β1 induced myofibroblasts lost their α-SMA expression when cultured in media containing serum [38]).

When TGF-β1 induced myofibroblasts were treated with adipose tissue conditioned media the proportion of α-SMA positive cells was significantly reduced. This implies a reversal or de-differentiation of the myofibroblast phenotype mediated by paracrine factors secreted from adipose tissue. The same effect was not seen with conditioned media from ADSC or lipid. Hoerst et al. [13] showed that conditioned medium from adipocytes caused de-differentiation of scar myofibroblasts which could indicate the effects we observed was a result of paracrine factors from the adipocyte component of whole fat.

To further validate this work, we repeated our experiments on HSF which were isolated from scar tissues and found the proportion of HSF expressing α-SMA was similar to TGF-β1 treated HDF. Adipose tissue conditioned medium did reduce the proportion of HSF which were α-SMA positive however the effect was not significant. In contrast, Hoerst et al. [13] was able to demonstrate a reduction in α-SMA protein expression in HSF following treatment with adipocyte conditioned media. Hoerst et al. used conditioned media from adipocytes differentiated from ADSCs which is likely to contain a different composition of paracrine factors when compared to our adipose tissue conditioned media.

Furthermore, differences observed between TGF-β1 treated HDF and HSF may reflect the clinical situation where established (older) scars have a reduced capacity to remodel [40–42]. Differences between HSF and TGF-β1 treated HDF have previously been described [16] and there will be heterogeneity in scar myofibroblasts both within and between patients [43]. The scar fibroblasts isolated in the study from Hoerst et al. were from excised hypertrophic and keloid scars that required surgical intervention. In our study HSF were isolated from scar tissue found in excised skin but these scars were not specifically undergoing scar revision surgery. It is possible the HSF in our study possessed a more established phenotype and that longer term treatment or a higher dose of paracrine factors may have been required to reduce the proportion of α-SMA positive HSF in this study compared to the scar fibroblasts used by Hoerst et al. [13]. While the effects in our study were not significant it is interesting that the difference between ADSC and adipose tissue conditioned media also held true in HSF.

There is a close relationship between the de-differentiation of myofibroblasts and proliferation. Previous work has shown that mitogens can trigger proliferation of myofibroblasts which resulted in apparent de-differentiation though the loss of stress fibres and downregulation of MyoD [37]. In our study the proportion of cells positive for α-SMA was used as the main outcome measure as it has been shown that the relative number of myofibroblasts and fibroblasts in a wound relates to the severity of the resulting scar [21, 40, 44]. An increased myofibroblast presence in wounds is associated with increased collagen deposition, more severe dermal scarring and increased patient morbidity [1, 2].

Treatment with conditioned media from adipose tissue led to an increase in cell number in the experiments where cells were treated with TGF-β1 before conditioned media was applied. This led us to the hypothesis that the conditioned media could be stimulating the proliferation of α-SMA negative fibroblasts, thus affecting the relative proportion of α-SMA cells in the overall culture. We showed that conditioned media from fat and ADSC both led to a (non-significant) increase in Ki-67 positive cells. When we measured the proportion of α-SMA positive cells which were also Ki-67 positive we found the majority of α-SMA positive cells were also Ki-67 positive demonstrating that the change in the proportion of myofibroblasts was not as a result of proliferation as both α-SMA positive and negative populations proliferated. This was also confirmed by the fact that conditioned media from ADSC and adipose tissue both increased the number of Ki-67 positive cells however only adipose tissue conditioned media affected the proportion of α-SMA positive cells.

## Conclusions

In this study we demonstrated that secreted factors from adipose tissue can inhibit myofibroblast differentiation and can induce the de-differentiation of myofibroblasts. Secreted factors from ADSC and lipid were not able to reduce the proportion of myofibroblasts, supporting the clinical use of whole adipose tissue over ADSC alone. While we have not been able to determine the mechanism through which adipose tissue reduces myofibroblast populations we have demonstrated that the effects observed are not solely due to ADSC, are not as a result of HGF in the conditioned media and are not caused by relative changes in proliferation. This study supports the use of autologous fat grafting for hypertrophic scar regeneration and demonstrates further investigation is needed to determine the mechanism.

### Supplementary Information

**Additional file 1**. Uncropped image of the western blot showing expression of α-SMA and GADH.

## Data Availability

The datasets generated during and/or analysed during the current study are available from the corresponding author on reasonable request.

## Abbreviations

ADSC: Adipose-derived stromal cells
DAPI: 4’,6-Diamindino-2-phenylindole
D-MEM: Dulbecco’s-modified Eagle’s medium
ECM: Extracellular matrix
FITC: Fluorescein isothiocyanate
GAPDH: Glyceraldehyde 3-phospate dehydrogenase
HGF: Hepatocyte growth factor
HDF: Human dermal fibroblasts
HSF: Human scar fibroblasts
PBS: Phosphate buffered saline
SVF: Stromal vascular fraction
TGF-β1: Transforming growth factor-β1
α-SMA: α-Smooth muscle actin
