# Supplementary figures and images for: Inhibition and reversal of a TGF-β1 induced myofibroblast phenotype by adipose tissue-derived paracrine factors
Source: Stem Cell Res Ther. 2024 Jun 13;15:166. doi: 10.1186/s13287-024-03776-3 (PMC11170827; doi:10.1186/s13287-024-03776-3)

### Anti- $\alpha$ -SMA stain

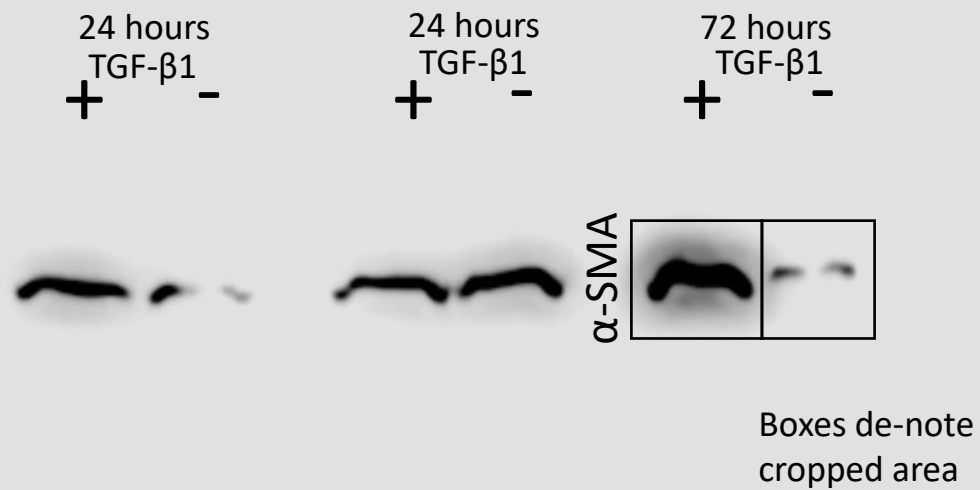

### Anti-GAPDH stain

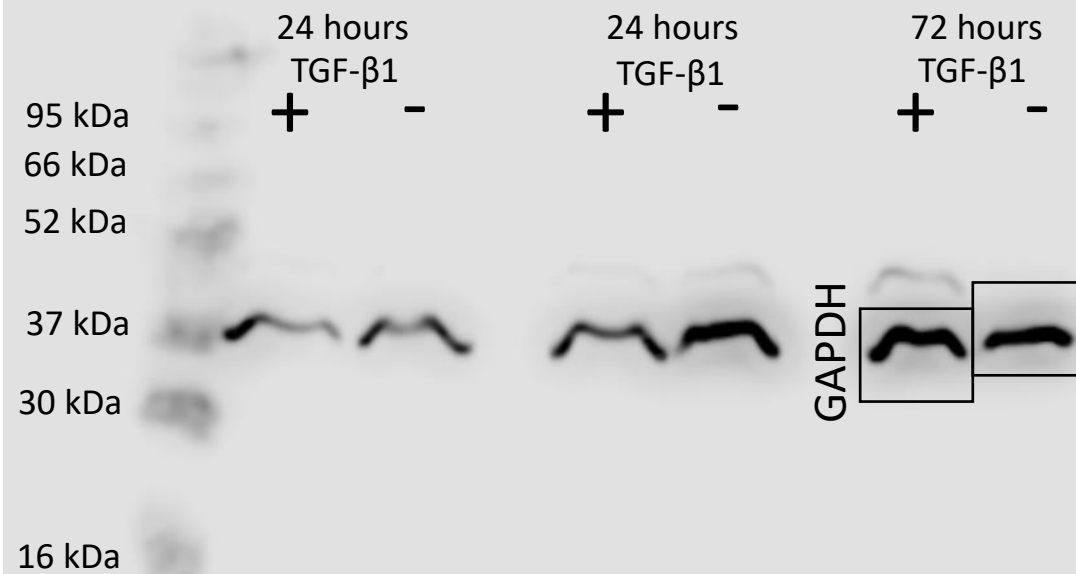

Supplement: Supplementary file 1 — Additional file 1. Uncropped image of the western blot showing expression of α-SMA and GADH. [file 13287_2024_3776_MOESM1_ESM.pdf]
